# Supplementary material for: Effect of Electrode Morphology on Performance of Ionic Actuators Based on Vat Photopolymerized Membranes
Source: Membranes (Basel). 2022 Nov 7;12(11):1110. doi: 10.3390/membranes12111110 (PMC9696670; doi:10.3390/membranes12111110)
Supplement: Supplementary file 1 [file membranes-12-01110-s001.zip › membranes-1975106-supplementary.pdf]

Supplementary Information for
Effect of Electrode Morphology on Performance of Ionic Actuators Based on Vat Photopolymerized Membranes

Oleg S. Morozov *, Anna V. Ivanchenko, Sergey S. Nechausov and Boris A. Bulgakov

Department of Chemistry, Lomonosov Moscow State University, 119991 Moscow, Russia
* Correspondence: osmorozov@yandex.ru

Table of contents

Figure S1. FTIR spectra for washed SWCNT/PVDF electrodes produced with different pore-forming agents 2

Figure S2. DSC curve for washed SWCNT/PVDF electrode made with PEG4000 2

Figure S3. DSC curve for washed SWCNT/PVDF electrode made with DBP 3

Figure S4. DSC curve for washed SWCNT/PVDF electrode made with $BMIMBF_4$ 3

Figure S5. Micrographs of electrodes and membranes connection 3

Figure S6. Complex plane plots of impedance spectra of actuators 4

Figure S7. Time dependence of current of the actuators under 3V during 5000 cycles at 0.4 Hz 4

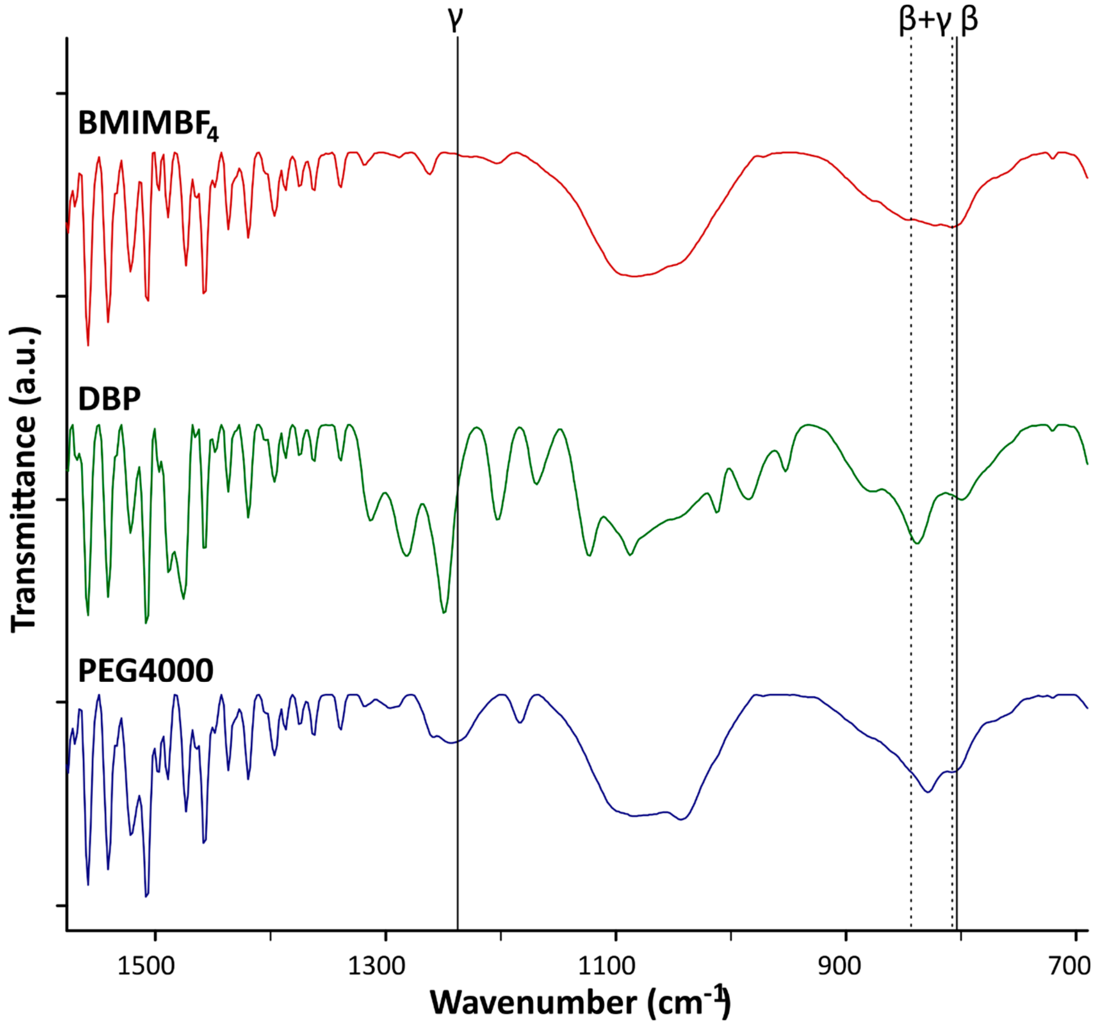

Figure S1. FTIR spectra for washed SWCNT/PVDF electrodes produced with different pore-forming agents

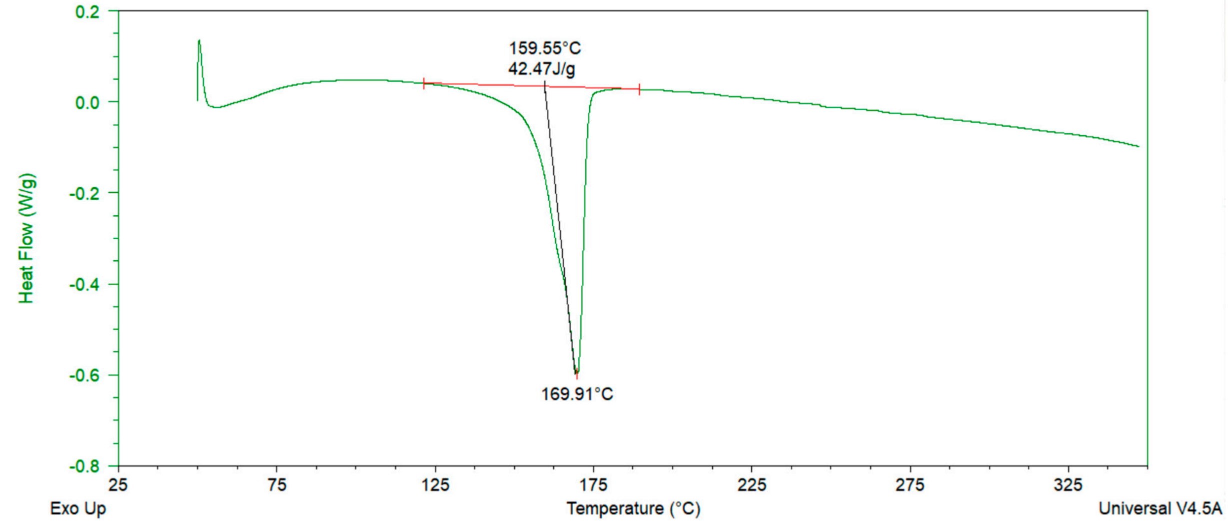

Figure S2. DSC curve for washed SWCNT/PVDF electrode made with PEG4000

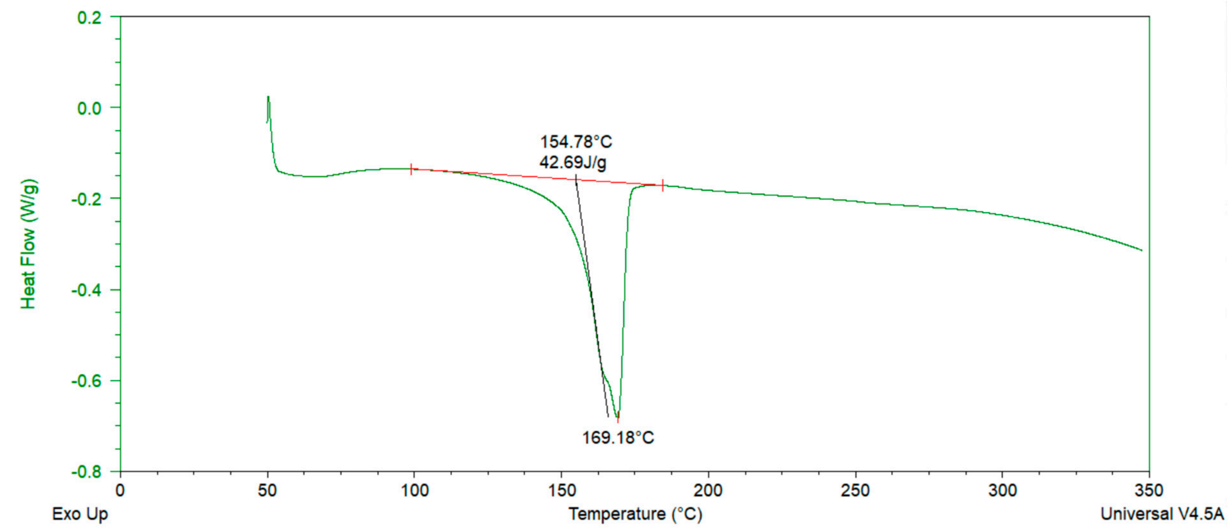

Figure S3. DSC curve for washed SWCNT/PVDF electrode made with DBP

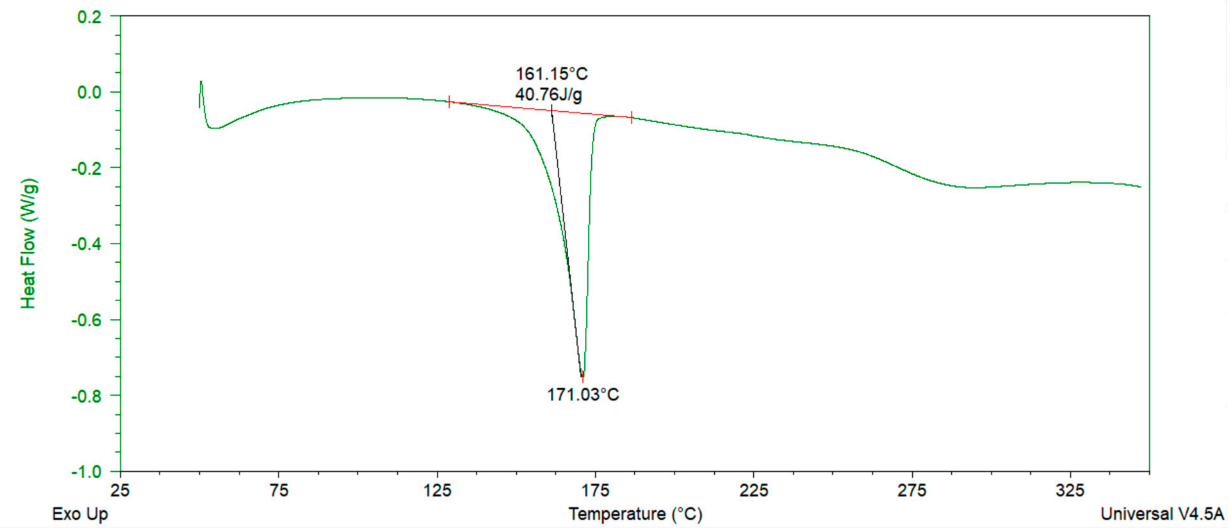

Figure S4. DSC curve for washed SWCNT/PVDF electrode made with BMIMBF$_4$

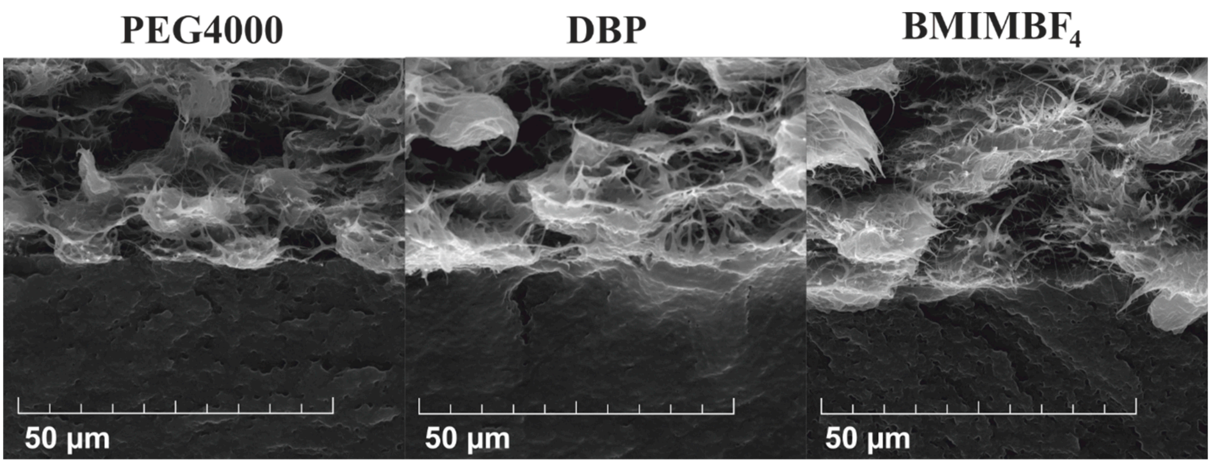

Figure S5. Micrographs of electrodes and membranes connection

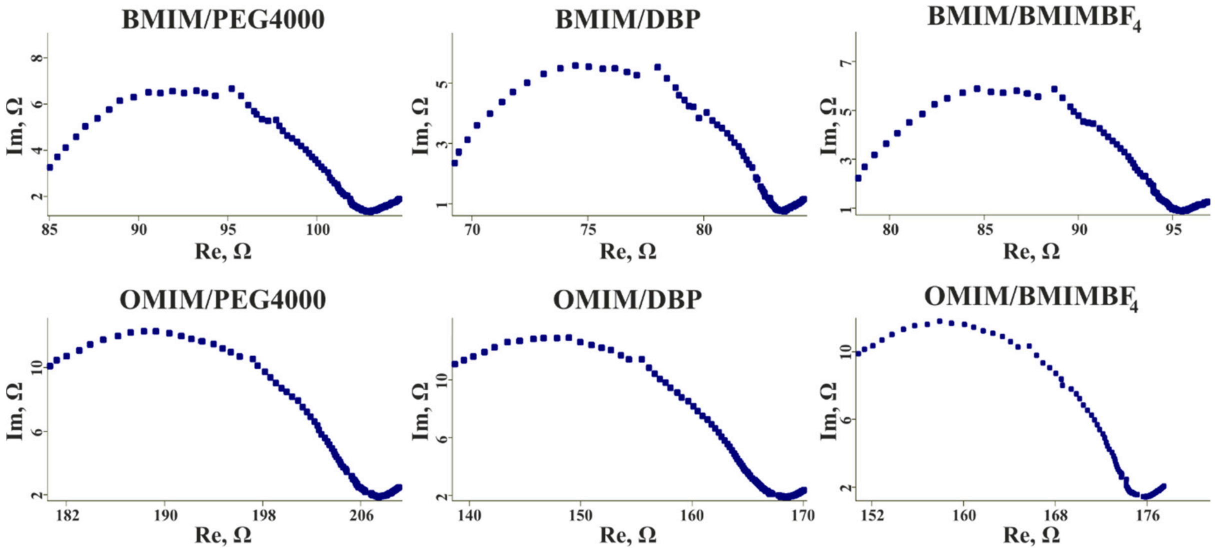

Figure S6. Complex plane plots of impedance spectra of actuators

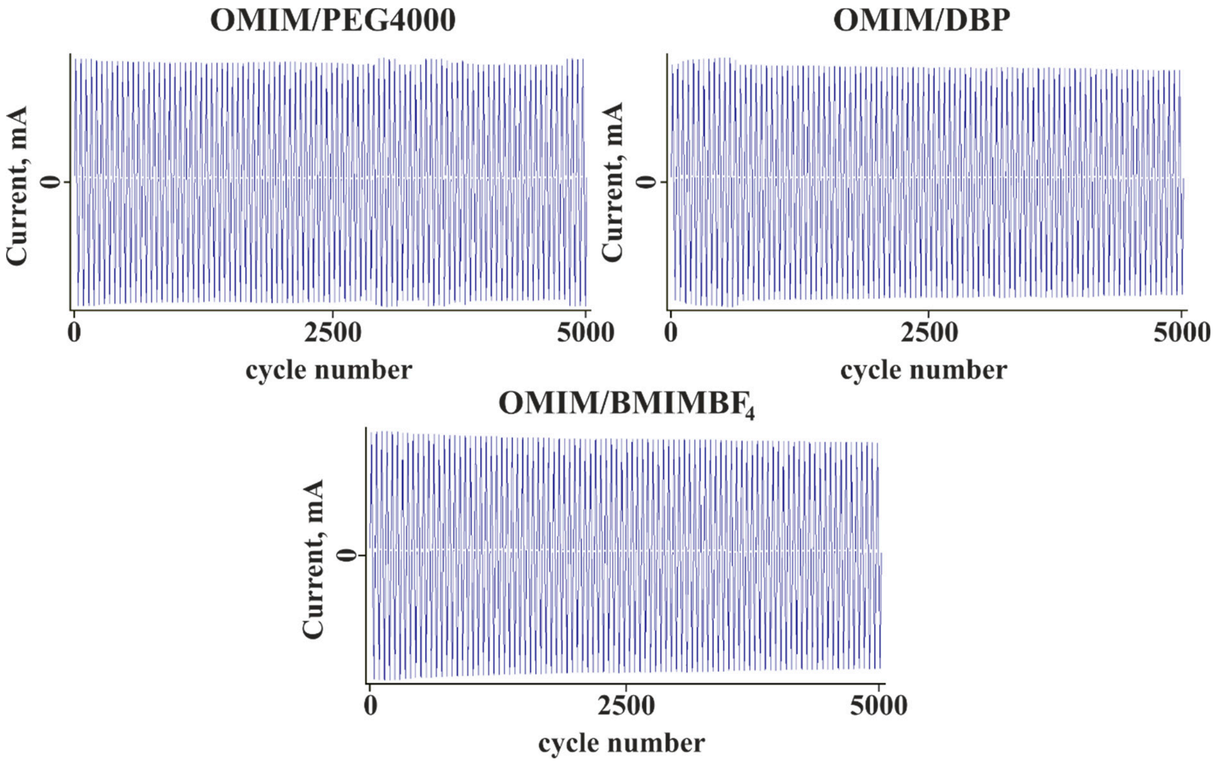

Figure S7. Time dependence of current of the actuators under 3V during 5000 cycles at 0.4 Hz
